# Correction: Association between *NF-kB* polymorphism and age-related macular degeneration in a high-altitude population

**DOI:** 10.1371/journal.pone.0271857

**Published:** 2022-07-18

**Authors:** Yan Xin, Kang Zefeng, Li Ling, Guan Ruijuan

There are errors in the Funding statement. The correct Funding statement is as follows: This work was supported by Department of science and technology of Qinghai Province (2017-ZJ-756); Key projects of Health Commission of Qinghai Province (2010-wjzd-02); Beijing Shijingshan District kangzefeng famous doctor inheritance studio project, Inheritance and innovation of traditional Chinese medicine "million million" talent project (Qihuang project) Qihuang scholars. The funders had no role in study design, data collection and analysis, decision to publish, or preparation of the manuscript.
