# Supplementary material for: Association between NF-kB polymorphism and age-related macular degeneration in a high-altitude population
Source: PLoS One. 2021 Jun 8;16(6):e0251931. doi: 10.1371/journal.pone.0251931 (PMC8186772; doi:10.1371/journal.pone.0251931)
Supplement: S1 File — (PDF) [file pone.0251931.s001.pdf]

项目编号：2017-ZJ-756

# 青海省科技计划项目
# 合同书

青海省科学技术厅制

## 三、项目人员

| 姓名 | 身份证号 | 所在单位 | 学历 | 职称 | 分工 | 投入时间（月） |
|---|---|---|---|---|---|---|
| 负责人 | | | | | | |
| 李凌 | 630102196306010045 | 青海省人民医院 | 学士 | 高级 | 项目负责人 | 30 |
| 项目参加人员 | | | | | | |
| 关瑞娟 | 142623198207043021 | 青海省人民医院 | 硕士 | 副高 | 项目主要完成任务 | 30 |
| 王笃亲 | 630104198302201540 | 青海省人民医院 | 硕士 | 副高 | 项目主要完成任务 | 20 |
| 张强 | 610113196912270512 | 青海省人民医院 | 硕士 | 高级 | 项目完成任务人 | 15 |
| 杨进寿 | 630104198001181531 | 青海省人民医院 | 学士 | 中级 | 项目完成任务人 | 15 |
| 秦志宏 | 630103197107120058 | 青海省人民医院 | 学士 | 副高 | 项目完成任务人 | 10 |
| 何玉清 | 632124197904131255 | 青海省人民医院 | 硕士 | 中级 | 项目完成任务人 | 10 |
| 冶占魁 | 632522197804040013 | 同德县人民医院 | 学士 | 初级 | 项目完成任务人 | 10 |
| 马雪英 | 220104196908051326 | 青海省人民医院 | 学士 | 高级 | 项目完成任务人 | 10 |
| 张蓉 | 630102197005300043 | 青海省人民医院 | 学士 | 副高 | 项目完成任务人 | 10 |
| 张晓英 | 632122197603140040 | 青海省人民医院 | 学士 | 副高 | 项目完成任务人 | 10 |

| 人员情况汇总 | | | | | | | |
|---|---|---|---|---|---|---|---|
| 总数（人） | 11 | 其中35岁以下研发人员 | 3 | 学科带头人 | 2 | | |
| 高级 | 7 | 中级 | 3 | 初级 | 1 | 其他 | 0 |
| 博士 | 0 | 硕士 | 4 | 学士 | 6 | 其他 | 0 |

非会员水印

**甲方（全称）：青海省科学技术信息研究所**

**乙方（全称）：青海省人民医院**

根据《中华人民共和国合同法》、《青海省省级财政科技专项资金管理办法》（青财教字〔2016〕2307号）、关于印发青海省重大科技专项等管理办法的通知》（青科发政〔2016〕164号）、《青海省科技计划和专项资金后补助管理办法》（青财教字〔2015〕2211号）等有关法律、规范性文件的规定，遵循自愿、公平和诚实信用的原则，双方就执行科技计划项目有关事项共同达成如下协议：

**一、项目概况**

1.1项目名称：高原地区老年性黄斑变性的流行病学研究

1.2项目计划类别：基础研究

1.3项目实施地点（具体到县区级）：西宁市、玉树州

**二、合同一般约定**

2.1合同范围

本文是根据法律规定和合同当事人约定具有约束力的文件，构成合同的文件包括合同条款及作为其附件的项目任务书。

2.2法律规范性文件

本合同所称法律是指中华人民共和国法律、行政法规、部门规章，以及项目所在地的地方性法规、自治条例、单行条例和地方政府规章等。

2.3合同所附项目任务书是本合同执行的主要内容，任务书中的要求内容是执行本合同的主要依据。

2.4联络

2.4.1甲方联络信息

甲方在本合同履行中接收文件的地点：西宁市城西区新宁路4号

甲方指定联系人：吴浩、李廷鹏

甲方指定的联系电话及传真号码：6117171，6302570，6145501（传真）

甲方指定的电子邮箱：qh6117171@163.com

2.4.2乙方联络信息

乙方在本合同履行中接收文件的地点：青海省人民医院（西宁市城东区共和路2号）

9.2.2 乙方因违反本合同约定收到甲方追回已拨付款项通知后30日内未退还全部已拨付财政科技专项资金的，甲方有权通过法律途径主张追回已拨付款项并要求乙方承担相应其他损失。

9.2.3乙方严重违反本合同约定且在项目申报、科研期间存在不诚信行为，或乙方严重违反合同约定导致项目研发失败后财政科技专项资金无法追回的，甲方将依据相关规定视情况将乙方单位、法定代表人、科研项目主要负责人列入信用青海联合惩戒黑名单，三年内取消申报科研项目的资格。

9.2.4因乙方违约导致甲方通过法律途径主张权利所发生的费用（包括但不限于诉讼费、保全费、担保费、律师代理费、差旅费等）由乙方承担。

**十、争议的解决**

10.1本合同履行中所发生的一切争议，双方应通过友好协商解决；如果协商不能解决，任何一方均可向甲方所在地有管辖权的人民法院提起诉讼。

**十一、其他事项**

11.1本合同任何条款被视为非法或不能执行并不影响本协议其他条款的效力。在特殊情况下，本合同无效和不能执行的条款应由各方相互协商，用能反映本合同各方真实意思能被各方合理接受的合法有效条款取代该无效或不能执行的条款。

11.2本合同仅为本合同双方的利益而订立，未经对方的事先书面同意，任何一方不能将其在本合同中所享有的权利和所承担的义务转让给任何第三方。

11.3本合同未尽事宜，各方可以按照公平合理的原则签订书面的补充协议；补充协议将构成本协议不可分割的组成部分。

11.4本合同的订立、效力、解释、履行受中华人民共和国法律的管辖。

11.5本合同所附的项目任务书以及各单位合作协议作为本合同的组成部分。

11.6本合同一式四份，每方各执二份。

甲方：青海省科学技术信息研究所

法定代表人（签章）：胡永强印

住所地：西宁市城西区新宁路4号

2017年9月11日

乙方：青海省人民医院

法定代表人（签章）：吴世政印

住所地：西宁市城东区共和路2号

2017年8月29日

# 医药卫生科技项目课题合同书（申报书）

项目名称：2016 年省卫生计生委指导性科研课题

课题名称：高原地区 CFH 基因 Y402H 基因多态与 AMD 易感性的相关性研究

课题申报单位：青海省人民医院

课题负责人：关瑞娟

起止年限：2016 年 9 月至 2018 年 9 月

青海省卫生和计划生育委员会

二○一六 年 七 月

## 2.课题负责人及主要参加人员

| 课题负责人 | | | | | | |
|---|---|---|---|---|---|---|
| 姓　名 | 性别 | 年龄 | 职务/职称 | 业务专业 | 为本课题工作时间（%） | 所在单位 |
| 关瑞娟 | 女 | 34 | 主治医师 | 眼科专业 | 90 | 青海省人民医院 |
| 李凌 | 女 | 53 | 科主任/主任医师 | 眼科专业 | 80 | 青海省人民医院 |
| 主要研究人员 | | | | | | |
| 何玉清 | 男 | 37 | 主治医师 | 眼科专业 | 50 | 青海省人民医院 |
| 张晓英 | 女 | 40 | 主治医师 | 眼科专业 | 50 | 青海省人民医院 |
| 张蓉 | 女 | 46 | 主治医师 | 眼科专业 | 50 | 青海省人民医院 |
| 朱金莲 | 女 | 42 | 主治医师 | 眼科专业 | 40 | 乐都县人民医院 |
| 杨金寿 | 男 | 36 | 主治医师 | 眼科专业 | 40 | 青海省人民医院 |
| 张道远 | 男 | 34 | 住院医师 | 眼科专业 | 30 | 青海省人民医院 |
| 马晓梅 | 男 | 41 | 副主任医师 | 眼科专业 | 30 | 青海省人民医院 |
| 秦志宏 | 男 | 45 | 主治医师 | 眼科专业 | 30 | 青海省人民医院 |
| 祁　恩 | 男 | 36 | 主治医师 | 眼科专业 | 30 | 青海省人民医院 |
| 王笃亲 | 女 | 34 | 主治医师 | 眼科专业 | 40 | 青海省人民医院 |
| | | | | | | |
| | | | | | | |

九、课题风险分析及对策

由于目前研究的人群（性别、年龄及种族）不同，样本量大小差异、检验方法不统一等原因，可能导致研究结论存在差异。我课题组使用在基因检测方面缺乏经验，因此在基因检测及统计学方面咨询专业人士，得到更多更可靠的数据。

十、有关附件

1．相关科研成果、专利等知识产权证明材料；

2．课题相关技术领域科技查新报告；

3．与课题相关的其他证明材料或文件等。

十一、本单位学术委员会意见（盖章）

青海省人民医院

年　　月　　日

十二、合同签订各方

主持部门（甲方）：

部门科技主管

技术负责人：刘芳

单位盖章

2017 年 3 月 3 日

承担单位（乙方）：

单位科技主管：

课题或合同负责人：

单位盖章

年 月 日

本合同自甲、乙双方签字、盖章之日起生效。

zow-wjzd-02

# 医药卫生科技项目课题合同书（申报书）

项目名称： 2020 年 卫 健 委 重 点 课 题

课题名称：滋阴明目汤治疗年龄相关性黄斑变性的机理研究

课题申报单位： 青海省人民医院

课题负责人： 李凌

起止年限： 2020 年 10 月至 2023 年 12 月

青海省卫生健康委员会

二 〇 二 〇 年 六 月

2.课题负责人及主要参加人员

| 课题负责人 | | | | | | |
|---|---|---|---|---|---|---|
| 姓　名 | 性别 | 年龄 | 职务/职称 | 业务专业 | 为本课题工作时间（%） | 所在单位 |
| 李凌 | 女 | 57 | 科主任/主任医师 | 眼科专业 | 90 | 青海省人民医院 |
| 关瑞娟 | 女 | 38 | 副主任医师 | 眼科专业 | 90 | 青海省人民医院 |
| 主要研究人员 | | | | | | |
| 晏鑫 | 男 | 24 | 在读硕士研究生 | 中医五官科学 | 70 | 青海大学医学院 |
| 张晓英 | 女 | 44 | 副主任医师 | 眼科专业 | 50 | 青海省人民医院 |
| 张蓉 | 女 | 50 | 副主任医师 | 眼科专业 | 50 | 青海省人民医院 |
| 杨进寿 | 男 | 40 | 主治医师 | 眼科专业 | 40 | 青海省人民医院 |
| 何玉清 | 男 | 41 | 主治医师 | 眼科专业 | 40 | 青海省人民医院 |
| 祁　恩 | 男 | 40 | 副主任医师 | 眼科专业 | 40 | 青海省人民医院 |
| 高添鹏 | 男 | 30 | 住院医师 | 眼科专业 | 50 | 青海省人民医院 |
| 汪亚萍 | 女 | 30 | 住院医师 | 眼科专业 | 50 | 青海省人民医院 |
| 王笃亲 | 女 | 38 | 副主任医师 | 眼科专业 | 40 | 青海省人民医院 |
| 张道远 | 男 | 38 | 副主任医师 | 眼科专业 | 40 | 青海省人民医院 |

十二、合同签订各方

主持部门（甲方）：

部门科技主管

技术负责人：

单位盖章

2020 年 9 月 8 日

承担单位（乙方）：

单位科技主管：

课题或合同负责人：

单位盖章

2020 年 9 月 2 日

本合同自甲、乙双方签字、盖章之日起生效。
