# Supplementary material for: Association between NF-kB polymorphism and age-related macular degeneration in a high-altitude population
Source: PLoS One. 2021 Jun 8;16(6):e0251931. doi: 10.1371/journal.pone.0251931 (PMC8186772; doi:10.1371/journal.pone.0251931)
Supplement: Attachment — Submitted filename: Response to reviewers.docx [file pone.0251931.s002.docx]

**April 9, 2021**

**Ravirajsinh Jadeja, Ph.D.**

**Academic Editor**

**PLOS ONE**

**Dear Dr. Jadeja,**

**Thank you for the opportunity to revise our manuscript. We have addressed all the issues raised by the Editor and both Reviewers. The following are the point-to-point responses to all comments.**

**Responses to the Editor:**

*When submitting your revision, we need you to address these additional requirements.*

*1. Please ensure that your manuscript meets PLOS ONE's style requirements, including those for file naming. The PLOS ONE style templates can be found at*

*https://journals.plos.org/plosone/s/file?id=wjVg/PLOSOne_formatting_sample_main_body.pdf and*

[*https://journals.plos.org/plosone/s/file?id=ba62/PLOSOne_formatting_sample_title_authors_affiliations.pdf*](https://www.editorialmanager.com/pone/about:blank)

**Response: We have revised our manuscript to meet PLOS ONE's style requirements.**

*2. PLOS ONE has specific criteria requiring that experiments, statistics, and other analyses are performed to a high technical standard and are described in sufficient detail (https://journals.plos.org/plosone/s/criteria-for-publication#loc-3). To that effect, please ensure that you have described who performed the methods and procedures and a more thorough discussion of how they were carried out, as well as further details regarding participant recruitment. If materials, methods, and protocols are well established, authors may cite articles where those protocols are described in detail, but your submission should include sufficient information to be understood independent of these references (https://journals.plos.org/plosone/s/submission-guidelines#loc-materials-and-methods).*

**Please note that PLOS ONE does not copy edit accepted manuscripts (https://journals.plos.org/plosone/s/criteria-for-publication#loc-5). To that effect, please ensure that your submission is free of typos and grammatical errors.*

**Response: We have revised the Methods according to PLOS ONE's requirements.**

*3. Thank you for stating the following in your Competing Interests section:*

*"I have read the journal's policy and the authors of this manuscript have the following competing interests: [insert competing interests here]"*

*Please complete your Competing Interests on the online submission form to state any Competing Interests. If you have no competing interests, please state "The authors have declared that no competing interests exist.", as detailed online in our guide for authors at http://journals.plos.org/plosone/s/submit-now*

*This information should be included in your cover letter; we will change the online submission form on your behalf.*

*Please know it is PLOS ONE policy for corresponding authors to declare, on behalf of all authors, all potential competing interests for the purposes of transparency. PLOS defines a competing interest as anything that interferes with, or could reasonably be perceived as interfering with, the full and objective presentation, peer review, editorial decision-making, or publication of research or non-research articles submitted to one of the journals. Competing interests can be financial or non-financial, professional, or personal. Competing interests can arise in relationship to an organization or another person. Please follow this link to our website for more details on competing interests:* [*http://journals.plos.org/plosone/s/competing-interests*](https://www.editorialmanager.com/pone/about:blank)

**Response: We have added the statement "The authors have declared that no competing interests exist" to the manuscript.**

*4. Please include your tables as part of your main manuscript and remove the individual files. Please note that supplementary tables (should remain/ be uploaded) as separate "supporting information" files*

**Response: We have included all tables as part of the main manuscript and have removed the individual files.**

*5. We note you have included a table to which you do not refer in the text of your manuscript. Please ensure that you refer to Table*

**Response: All tables have been referred to within the text of the revised manuscript.**

**Responses to Reviewer #1:**

*Reviewer #1: Yin et al examined 55 patients and 57 controls from the Qinghai Provincial People’s Hospital and genotyped them for four SNPs in NFK-B. The paper claims a risk association with NFK-B for risk of AMD in a population that had lived more than 20 years in a high-altitude climate (> 2000 m). The study is interesting but there are some major issues.*

**Response: Thank you for the opportunity to improve our manuscript.**

*1) The usual age of diagnosis of AMD is > 60. However, here > age 40 was used. Could the authors please clarify?*

**Response: We agree with the Reviewer that the use of ≥ 40 years of age as an inclusion criterion for AMD cases and controls may have compromised the representativeness of the samples in this study. Although AMD can be diagnosed as early as 35 years, most AMD cases are diagnosed at ≥ 60 years [Song P, Du Y, Chan KY, Theodoratou E, Rudan I. The national and subnational prevalence and burden of age-related macular degeneration in China. J Glob Health. 2017 Dec;7(2):020703.]. In addition, since control participants younger than 60 years may develop AMD later in life, our inclusion criterion of patients ≥ 40 years may have introduced selection bias in this study, resulting in lower power to detect real associations. We have added these statements to the Discussion.**

*2) The sample size is extremely small (slightly over 100 people), and therefore conclusions regarding age, gender, or other differentiation are extremely hard to ascertain, including association of NFK-B to AMD. Could the authors provide please how they were able to have the power to draw the conclusions they did?*

**Response: We agree with the Reviewer that the power of our study to detect the association between the *NF-kB* gene polymorphisms and AMD was limited due to the small sample size. We estimated that this study had the power of 78% to detect the association at OR = 2.0 and the prevalence at 7.11% for people aged 65–69 years [Song P, Du Y, Chan KY, Theodoratou E, Rudan I. The national and subnational prevalence and burden of age-related macular degeneration in China. J Glob Health. 2017 Dec;7(2):020703.]. Therefore, the negative associations we observed after correcting for multiple tests in this study did not disprove potential associations between the *NF-kB* gene polymorphisms and AMD. We have added these statements to the Discussion.**

*3) No comparison to non-high-altitude patients was obtained, even a larger study to evaluate the NFK-B polymorphism to see how common the alleles are in the regular population- which means that this study cannot come to the conclusion as to whether or not the alternate allele is associated with AMD in the high-altitude population alone. The reader does not even know how common the alternate alleles are in the general population through examining databases like GnomeAD or ExAC. Could the authors please clarify?*

**Response: One of the strengths of this study is that all patients with AMD were long-term (> 20 years) residents at a high altitude (> 2,000 m), which is a unique population that is under-represented in genetic studies of AMD. However, we agree that the lack of comparison to non-high-altitude patients in this study made it impossible to conclude that the risk alleles are associated with AMD in the high-altitude population alone. The allele frequencies of the four SNPs analyzed in this study are comparable to the corresponding allele frequencies in East Asians reported in the public gnomAD database (Table 3). We have added these statements to the Discussion.**

*4) The p-values are extremely high (0.04) to declare an association.*

**Response: We agree that the associations we observed in this study were nominally significant and did not survive the Bonferroni correction (corrected *P* > 0.05). We have clarified this in the Results.**

*5) The paper language needs to be edited along with spelling and grammar, to improve the clarity for the reader.*

**Response: Thank you for the comments. We have improved our language by correcting spelling and grammar.**

**Responses to Reviewer #2:**

*Reviewer #2: Association between NF-kB polymorphism and age-related macular degeneration in a high-altitude population.*

*The authors have intended to investigate the association between NF-kB gene polymorphism and age-related macular degeneration in a high-altitude population. The study concludes that the AA genotype at rs3774932 and CC genotype at rs3774937 in NF-kB gene could be the risk genotypes for AMD. However, this reviewer has few major concerns to be addressed by the authors in order to enhance the quality of the manuscript.*

*• The ethical committee numbers are different on the ethics statement and in the manuscript.*

**Response: Thank you for pointing out this error. We have corrected it in the manuscript.**

*Introduction: Overall, this section needs to be revised.*

**Response: We have revised the Introduction.**

*• The details in this section are not sufficient enough to substantiate the primary goal. I suggest including more information and citations relevant to the Nf-KB gene and its association with AMD.*

**Response: We have added more information and citations relevant to the *NF-kB* gene and its association with AMD.**

*• The subheading “Subjects” could be under the methods section.*

**Response: We have revised the subheadings across the entire manuscript.**

*Methods: Overall, the methods section needs a revision and more references could be added to enable the readers to follow without confusion.*

*• 1.2.1 - Provide sample size with more details.*

*• 1.2.2 - Provide reference and add more details.*

*• 1.2.3 - Provide the primers and probe details in the table format.*

*• 1.2.4 - The representative gel images could be added.*

*• 1.2.5 - Very less details*

**Response: We have revised the Methods according to the Reviewer’s comments.**

*• Did the author further confirm the genotypes/SNP of these four variants of NF-KB by PCR-RFLP?*

**Response: We did not further confirm the genotypes of these four SNPs in *NF-kB* by PCR-RFLP. However, we used the well-known MassARRAY system to genotype the SNPs.**

*• Besides, either the ChIP assay to identify the binding activity of these genotypes or luciferase reporter assay to test the function of these polymorphisms, especially the NF-κB binding site activity in AA and CC alleles, could have been attempted.*

**Response: Thank you for the comments on functional studies. Those will be one of the major aims in our future studies.**

*Results: Overall, the results are not presented well and need thorough revision.*

**Response: We have thoroughly revised the Results section.**

*• 2.3 - grou-----group*

**Response: We have corrected this typo.**

*Discussion:*

*The current data obtained from this study was not sufficient enough to conclude. Also, the findings are not discussed well.*

*• The results should be discussed with respect to how these genetic variations (rs3774932 and rs3774937) might influence gene transcriptional regulation and expression, consequently affecting NF-κB pathway activation and/or the susceptibility to AMD pathology.*

**Response: We have revised the Discussion.**

*Figures &Tables:*

*Some of the details on table-3 and supplementary materials are provided in a different language.*

**Response: We have removed all non-English text from the manuscript.**

*Please consider.*

*• The punctuations/ spacings throughout the manuscript should be rectified.*

**Response: We have reformatted the entire manuscript.**
